# Plasma- and Saliva Exosome Profile Reveals a Distinct MicroRNA Signature in Chronic Periodontitis

**DOI:** 10.3389/fphys.2020.587381

**Published:** 2020-11-30

**Authors:** Nik Nur Syazana Nik Mohamed Kamal, Raja Azman Raja Awang, Suharni Mohamad, Wan Nazatul Shima Shahidan

**Affiliations:** School of Dental Sciences, Universiti Sains Malaysia, Health Campus, Kota Bharu, Malaysia

**Keywords:** biomarker, inflammation, profiling, miRNA – microRNA, exosome

## Abstract

Chronic periodontitis (CP) is an oral cavity disease arising from chronic inflammation of the periodontal tissues. Exosomes are lipid vesicles that are enriched in specific microRNAs (miRNAs), potentially providing a disease-specific diagnostic signature. To assess the value of exosomal miRNAs as biomarkers for CP, 8 plasma- and 8 salivary-exosomal miRNAs samples were profiled using Agilent platform (comparative study). From 2,549 probed miRNAs, 33 miRNAs were significantly down-regulated in CP as compared to healthy plasma samples. Whereas, 1,995 miRNAs (1,985 down-regulated and 10 up-regulated) were differentially expressed in the CP as compared to healthy saliva samples. hsa-miR-let-7d [FC = −26.76; AUC = 1; *r* = −0.728 [*p*-value = 0.04]), hsa-miR-126-3p (FC = −24.02; AUC = 1; *r* = −0.723 [*p*-value = 0.043]) and hsa-miR-199a-3p (FC = −22.94; AUC = 1; *r* = −0.731 [*p*-value = 0.039]) are worth to be furthered studied for plasma-exosomal samples. Meanwhile, for salivary-exosomal samples, hsa-miR-125a-3p (FC = 2.03; AUC = 1; *r* = 0.91 [*p*-value = 0.02]) is worth to be furthered studied. These miRNAs are the reliable candidates for the development of periodontitis biomarker, as they were significantly expressed differently between CP and healthy samples, have a good discriminatory value and strongly correlate with the mean of PPD. These findings highlight the potential of exosomal miRNAs profiling in the diagnosis from both sourced as well as provide new insights into the molecular mechanisms involved in CP.

## Introduction

Periodontitis is an inflammation disease (Suzuki et al., 2004; Kato et al., 2013), characterized by gingival swelling, loss of alveolar bone and movement of the teeth. Chronic periodontitis (CP) is a slow progression destructive type of periodontal disease (Papapanou et al., 2018). The CP disease detection still relies on conventional clinical measurements: charting dental plaque accumulation, measuring periodontal pocket depth (PPD), the calculation of percentage of bleeding on probing (BOP) and radiographic detection of alveolar bone loss (ABL). The outcome of these evaluations can vary greatly depending on the dentists’ skills and experience (Fujimori et al., 2019). Hence having a quantitative method might be a good alternative approach in having a stable and objective diagnosis for the disease.

miRNAs are non-coding ribonucleic acid (RNA) molecules (20–25 nucleotides), acting as post-transcriptional regulators by binding to the 3′ untranslated regions (3′-UTRs) of target messenger RNAs (mRNAs). They have negative regulation of gene expression (degrading target mRNA or inhibit the translation of protein product) (Lee et al., 2011) and have been well characterized to play a role in cell growth, differentiation, apoptosis, pathogen-host interactions, stress responses and immune function (Lu et al., 2005; Stadler and Ruohola-Baker, 2008). miRNAs have been proposed as excellent salivary biomarker candidates due to their ease of isolation and identification through quantitative PCR (Michael et al., 2010).

Several miRNAs studies had been carried out in finding suitable biomarker for the quantitative method in diagnosing the CP disease. Seven of them used gingival tissues as a source of the miRNAs (Lee et al., 2011; Xie et al., 2011; Perri et al., 2012; Stoecklin-Wasmer et al., 2012; Ogata et al., 2014; Motedayyen et al., 2015; Li et al., 2019). However, with the emerging popularity in finding non-invasive type of biomarker (Wang et al., 2015), the study now had shifted towards gingival crevicular fluid (GCF) (Saito et al., 2017) and saliva (Fujimori et al., 2019) as miRNAs source. As saliva collection do not require the present of professional staffs, e.g., phlebotomist or medical doctor; and with the future aim in developing self-collect saliva-test kit, the use of saliva for tests had come to the light of attention. One of the recent publication on miRNAs as CP biomarker is a salivary-exosomes study carried out by Fujimori’s group (2019). They profiled 84 miRNAs and found only hsa-miR-381-3p to show a significant difference between no/mild periodontitis as compared to the severe periodontitis group. However, validation of hsa-miR-381-3p using quantitative real-time polymerase chain reaction (qPCR) assays showed insignificant difference. They stated that one of their study limitations was the number of miRNAs tested. They concluded that there was a possibility of other non-tested miRNAs that can be a potential biomarker for the CP disease (Fujimori et al., 2019).

In this study, we used Agilent platform with updated database-consisting of 2,549 miRNAs. Thus, we believed that more findings could be produced in addition to the previous one. Furthermore, the profiling of plasma exosomal miRNAs was also compared with salivary exosomal miRNAs profile. This comparison is hoped to provide useful insights in exploring potential biomarkers for CP diagnostics.

## Materials and Methods

### Recruitment and Clinical Examination

Eight CP and eight healthy individuals were recruited from Dental Clinic, Universiti Sains Malaysia (USM), Health Campus, Kelantan, Malaysia. For each individual, PPD, BOP, plaque score, presence of radiographic evidence of ABL and presence of gingivitis were examined using calibrated periodontal probes (full mouth periodontal evaluation; 6 sites per tooth: mesio-buccal, buccal, disto-buccal, mesio-lingual, lingual, and disto-lingual). Inclusion criteria for CP individuals: have ≥ eight sites in different tooth with PD ≥ 5 mm, BOP ≥ 20%, and radiographic evidence of ABL. Inclusion criteria for healthy individuals: have PD < 3 mm, BOP ≤ 20%, and no radiographic evidence of ABL. Exclusion criteria: age < 25 years old, have original teeth < 15 (CP) and 24 (healthy), smoking, pregnant/breast-feeding, have any systemic diseases, e.g., diabetes mellitus type (uncontrolled), or allergy, have serious heart, liver or kidney problems, been involved in any organ transplant, on medication that affect periodontal tissues, and/or having trauma or tooth extraction 2 weeks prior to sampling.

### Samples Collection and Processing

Each individual was asked to refrain from eating, drinking, using oral hygiene products, and to rinse their mouth with water for at least 1 h prior to sample collection. Unstimulated saliva was collected at random time on the same day as oral examination. Saliva was collected about 10 ml (or within 20 min) into sterile sample collection container. For blood collection, 10 ml peripheral blood was collected into Ethylenediaminetetraacetic acid (EDTA) tube. The blood was centrifuged at 2,000 × *g* at 4°C for 15 min for plasma extraction. The saliva was centrifuged at 6,000 × *g* for 20 min at room temperature to eliminate food debris and cells. Exosomes were isolated by ultracentrifugation [110,000 × *g* at 4°C for 2 h, Sorvall RC-100, United States; according to Kechik et al.’s (2018) protocol] of 4 ml plasma or 1.5 ml saliva from each individual. The total RNA was extracted using miRNeasy micro kit (Qiagen, United States) according to manufacturer’s protocol, and quantified using NanoDrop (Thermo Fisher Scientific, United States). The quality of total RNA and the presence of miRNAs were confirmed using RNA Nano 6000 chip and small RNA chip (Agilent Technologies, United States), respectively.

### JESS Automated Western Blot Analysis

Protein from each sample was extracted using RIPA lysis buffer (Biotechnology Grade; VWR Life Science, United States) and their concentration was quantified using Bradford assay (Bio-Rad, United States), at 600 nm. The automated western blot was run according manufacturer’s protocol (JESS’s assay module Protein Normalization: AM-PN01), using 25 capillary cartridges (R&D Systems, United States: part number = SM-PN01-1). About 3 μg of protein was used per sample, per marker: CD9 (Novus Biologicals, United States: catalog number = NBP2-2217), CD63 (R&D Systems, United States: catalog number = MAB50482), Hsp70 (R&D Systems, United States: catalog number = MAB1663) and CD81 (Novus Biological, United States: catalog number = NB100-65805). The proteins were detected and analyzed using Compass for Simple Western software (United States).

### Transmission Electron Microscopy Analysis (TEM)

The isolated exosomes were diluted with dilution factor 1:1000 in sterile 1× phosphate buffered saline (PBS). Approximately, 5 μl of diluted exosomes elution was dropped on Formvar-carbon coated EM grids and left aside for 20 min to allow the absorption of exosomes into membranes. These exosomes-coat grids were fixed with 0.6% glutaraldehyde for 4 min and then washed twice with distilled water for 1 min, each. The grids were stained with 2% uranyl acetate at pH 7 for 5 min. Finally, the grids were viewed using transmission electron microscope at a voltage of 80 kV. Digital images with scale bar were captured in magnification in range of 10,000–160,000.

### MiRNAs Profiling and Statistical Analysis

MiRNAs profiling was performed using human miRNA microarray 8 × 60 k kit (Agilent Technologies, United States). The starting material for each sample was set to the lowest concentration of each set: 30 ng for plasma and 53 ng for saliva samples. Data were analyzed using GeneSpring GX software (Agilent Technologies, United States). The moderated *t*-test with Benjamin-Hochberg multiple testing corrections were applied. The differences were considered significant when the probability value was less than 5% (*p* < 0.05), and fold change was more than 2.0 (FC > 2).

### Bioinformatics

Target genes of selected differential miRNAs were predicted using the TargetScan database^1^. These genes were further overlap with findings in previous CP miRNAs studies.

### Statistics

SPSS statistics version 26.0 (IBM, United States) was used for statistical analysis. The data between CP and healthy individuals were determined using either Student’s *t*-test or Chi-square test. Receiver Operating Characteristics (ROC) curves and area under curve (AUC) were calculated to determine the specificity and sensitivity of the selected miRNAs as potential CP biomarkers. A Pearson’s correlation test was carried out to analyze the relationship between the candidate miRNAs and clinical parameters (mean of PPD). *P*-value ≤ 0.05 was considered a statistically significant difference.

## Results

### Samples Collection and Processing

Two independent sets of (mean age 49.0 and 47.0) and healthy (mean age 30.5 and 26.3) individuals were recruited for this plasma and saliva study, respectively (Table 1). RNA Nano 6000 electropherograms showed peaks for small RNAs (size < 200 nucleotides); while small RNA electropherograms showed peaks for miRNAs (size < 30 nucleotides) (Supplementary Figure S1). These observations suggested that the RNA isolation procedure was successful in collecting miRNAs. No ribosomal RNA peaks (at 40 to 50 s) were observed in RNA Nano electropherograms (Supplementary Figure S1), indicating that exosomal samples were not contaminated with RNA derived from the cellular fraction.

**TABLE 1:** Characteristics of recruited individuals and their samples’ total RNA and miRNAs concentration.

Samples	Age	G	Total teeth	≥8 sites with PPD ≥5 mm and ^##^Bone loss	Medical problem	Systemic disease	Nanodrop	Nano 6000 chip	Small RNA chip
		
							Total RNA (ng/μl)		miRNA (pg/μl)
**#Set1: Plasma-exosomal samples (age range 26–62 years old)**									
CP1	57	F	24	Yes (5.3)^€^	Yes^2a^	No	48	5	155.2
CP2	45	F	23	Yes (5.3) ^€^	No	No	25	3	86.9
CP3	62	F	17	Yes (6.5) ^€^	Yes^2b^	No	15	6	1.5
CP4	32	F	30	Yes (5.6) ^€^	No	No	38	4	112.4
H1	24	F	>20^ε^	No	No	No	22	3	89.3
H2	28	F	>20 ^ε^	No	No	No	34	2	143.3
H3	27	F	>20 ^ε^	No	No	No	38	11	96.6
H4	42	F	>20 ^ε^	No	No	No	32	4	423.4
*p*-value	0.053^¶^	-	0.019^¶^		0.430^¥^	1.000^¥^	1.000^¶^	0.823^¶^	0.291^¶^
**#Set2: Salivary-exosomal samples (age range 26–51)**									
CP5	41	F	31	Yes (5.7) ^€^	No	No	35	11	28,854.8
CP6	49	F	28	Yes (5.3) ^€^	No	No	35	30	21,306.5
CP7	45	F	17	Yes(5.5) ^€^	Yes^1a^	Yes^1b^	98	64	99,230.2
CP8	46	M	25	Yes (5.4) ^€^	Yes^1c^	No	34	28	30,684.6
H5	26	M	>20 ^ε^	No	No	No	42	3	1,190.7
H6	26	F	>20 ^ε^	No	No	No	27	1	1,082.5
H7	26	F	>20 ^ε^	No	No	No	48	7	13,232.3
H8	27	M	>20 ^ε^	No	No	No	46	3	12,143.7
*p*-value	<0.001^¶^	1.000^¥^	0.070^¶^		0.430^¥^	1.000^¥^	0.580^¶^	0.037^¶^	0.085^¶^

*CP = chronic periodontitis individual sample; H = healthy individual sample; G = gender; F = female; M = male; PPD = periodontal pocket depth; ≥ = have more than; ^##^Bone loss = radiographic evidence showing alveolar bone loss; Nano 6000 chip = RNA Nano 6000 chip. Yes^1a^ = controlled hypercholesterolemia and hypertension (on medication); Yes^1b^ = controlled diabetes mellitus (on medication). Yes^1c^and Yes^2a^ = controlled hypertension (on medication); Yes^2b^ = controlled hypertension and hyperlipidemia (on medication). ^¶^ = p-value (CP vs. H) that calculated using t-test; ^¥^ = p = value (CP vs. H) that calculated using Chi-square test; ^€^ = average periodontal pocket depth; ^ε^ = in calculating p-value, the original teeth of healthy individuals were assumed to be 32. This is because the condition was checked but not recorded.*

### JESS Automated Western Blot Analysis

**FIGURE 1:**
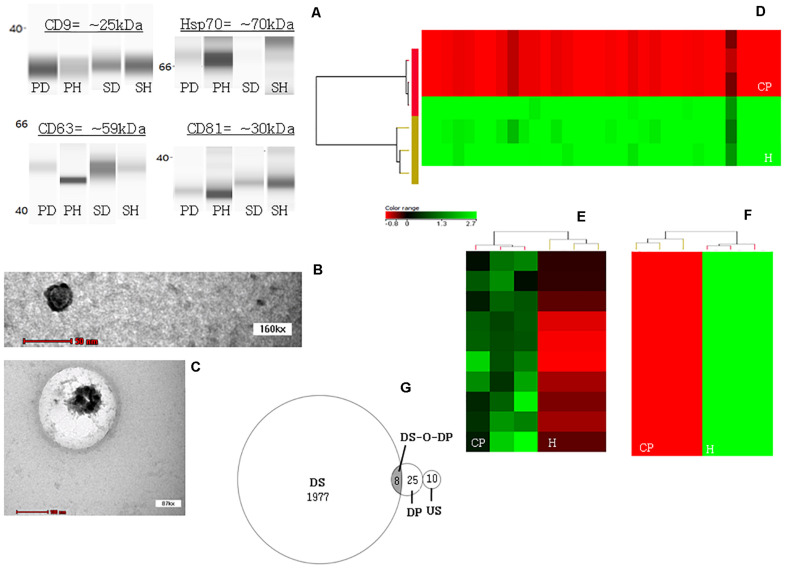
JESS automated western blotting analysis, transmission electron microscopy (TEM) analysis and profiling heatmaps. **(A)** Confirmation that ultracentrifugation pellet contains exosomes. The JESS automated western blot analysis shows the detection of specific exosomal markers CD9, CD63, Hsp70, and CD81 in both plasma- and salivary-exosomal samples except for the Hsp 70 from CP salivary-exosomal representative sample showed faint band. [PD = plasma diseased sample/CP plasma-exosomal representative sample; PH = plasma healthy/healthy plasma-exosomal representative sample; SD = saliva diseased/healthy salivary-exosomal representative sample; and SH = saliva healthy/healthy salivary-exosomal representative sample]. **(B)** TEM image of a single exosome originated from healthy plasma-exosomal representative sample. **(C)** TEM image of a single exosome originated from healthy salivary-exosomal representative sample. **(D)** Heatmap illustrating all 33 significantly down-regulated miRNAs in CP as compared to healthy plasma-exosomal samples. **(E)** Heatmaps illustrating all significantly up-regulated miRNAs in CP as compared to healthy salivary-exosomal samples. **(F)** Heatmaps illustrating top ten significantly down-regulated miRNAs in CP as compared to healthy salivary-exosomal samples. Color shading reflects expression level [color version = with red indicating low expression and green indicating high expression; black-and white version = with darker black indicating low expression and white or greyer color indicating high expression]. **(G)** Venn diagram shows the distribution of total miRNAs that are down-regulated only in plasma- (DP) and in salivary- (DS) exosomal, up-regulated in only salivary-exosomal (US), and down-regulated in both plasma- and salivary-exosomal (DS-O-DP) CP samples as compared to their respective exosomal healthy samples.

Expression of exosomal markers CD9, CD63, Hsp70, and CD81 were detected from both plasma- and salivary-exosomal samples except for the Hsp 70 from CP salivary-exosomal representative sample showed faint band (Figure 1A).

### Transmission Electron Microscopy Analysis (TEM)

A single exosome was successfully viewed under TEM at 160 kx using healthy plasma-exosomal representative sample (Figure 1B), while another single exosome was successfully viewed under TEM at 87 kx using healthy salivary-exosomal representative sample (Figure 1C).

### MiRNAs Profiling Analysis

Table 2A and Figure 1D shows all the 33 significantly down-regulated miRNAs in CP as compared to healthy plasma-exosomal samples. Table 2B and Figures 1E,F shows all ten significantly up-regulated and top ten significantly down-regulated miRNAs (from total of 1,985 down-regulated miRNAs), in CP as compared to healthy salivary-exosomal samples. The heatmap of the top ten significantly down-regulated miRNAs showed clear contrast as compared to the heatmap of 10 significantly up-regulated miRNAs (Figures 1E,F). Eight miRNAs were significantly down-regulated in both plasma- and salivary-exosomal samples: hsa-let-7d-5p, hsa-miR-103a-3p, hsa-miR-126-3p, hsa-miR-150-5p, hsa-miR-199a-3p, hsa-miR-4485-5p, hsa-miR-6088, and hsa-miR-6821-5p (Figure 1G and Table 2C).

**TABLE 2:** *P*-value, fold change (FC) and area under curve (AUC) of miRNAs in CP exosomal samples as compared to healthy exosomal samples are tabulated as below.

miRNAs	Corrected *p*-value	FC	AUC	miRNAs	Corrected *p*-value	FC	AUC	
**(A) #Set1: plasma samples (all significantly down-regulated miRNAs, without Benjamin-Hochberg multiple testing corrections)**								
hsa-let-7b-5p	2.65E-05	−112.73	1.00	hsa-let-7i-5p	5.91E-05	−33.57	1.00	
hsa-miR-24-3p	3.74E-05	−74.12	1.00	hsa-miR-19b-3p	0.002	−32.13	1.00	
hsa-miR-6821-5p	5.91E-05	−54.17	1.00	hsa-miR-3135b	0.002	−30.21	1.00	
hsa-miR-21-5p	1.38E-05	−48.23	1.00	hsa-miR-320e	3.61E-04	−29.96	1.00	
hsa-miR-4485-5p	0.001	−47.05	1.00	hsa-miR-22-3p	2.95E-06	−28.89	1.00	
hsa-miR-3663-3p	0.010	−45.62	0.83	hsa-let-7d-5p	1.61E-04	−26.76	1.00	
hsa-miR-92a-3p	0.002	−44.71	1.00	hsa-miR-23a-3p	1.61E-04	−25.98	1.00	
hsa-miR-320d	0.001	−44.47	1.00	hsa-miR-15b-5p	2.98E-04	−24.34	1.00	
hsa-miR-6850-5p	5.55E-05	−43.60	1.00	hsa-miR-126-3p	2.98E-04	−24.02	1.00	
hsa-miR-6088	3.11E-04	−42.38	1.00	hsa-miR-199a-3p	1.84E-04	−22.94	1.00	
hsa-miR-20a-5p	1.71E-07	−42.00	1.00	hsa-let-7g-5p	5.59E-06	−21.39	1.00	
hsa-let-7a-5p	4.82E-05	−41.20	1.00	hsa-miR-17-5p	2.86E-05	−20.89	1.00	
hsa-miR-197-3p	2.98E-04	−40.43	1.00	hsa-let-7f-5p	5.59E-06	−19.27	1.00	
hsa-miR-766-3p	2.13E-04	−39.57	1.00	hsa-miR-25-3p	5.59E-06	−18.60	0.50	
hsa-miR-2861	0.002	−35.80	1.00	hsa-miR-150-5p	0.005	−17.14	0.89	
hsa-miR-320c	4.33E-04	−34.28	1.00	hsa-miR-6090	0.005	−5.627	1.00	
hsa-miR-103a-3p	3.55E-04	−33.96	1.00					

**(B) #Set2: saliva samples (all significantly up-regulated and ten most significantly down-regulated miRNAs).**								

hsa-miR-5006-5p	0.002	4.85	1.00	hsa-miR-942-3p	0.011	−409.74	0.67	
hsa-miR-7108-5p	0.031	4.06	1.00	hsa-miR-936	0.010	−379.77	0.67	
hsa-miR-575	0.004	3.42	0.67	hsa-miR-4485-5p	0.010	−372.05	0.47	
hsa-miR-6786-5p	0.034	3.31	1.00	hsa-miR-4664-3p	0.009	−351.18	0.50	
hsa-miR-4793-3p	2.48E-06	3.25	0.83	hsa-miR-4485-3p	0.008	−348.23	0.67	
hsa-miR-6824-5p	0.003	3.16	1.00	hsa-miR-629-3p	0.007	−325.38	0.67	
hsa-miR-4440	7.71E-06	3.03	1.00	hsa-miR-1287-5p	0.006	−299.26	0.67	
hsa-miR-1224-5p	0.032	2.10	1.00	hsa-miR-378i	0.006	−294.15	0.67	
hsa-miR-320a	0.0049	2.07	1.00	hsa-miR-6073	0.004	−258.89	0.67	
hsa-miR-125a-3p	0.038	2.03	1.00	hsa-miR-208a-5p	0.003	−236.75	0.50	

**(C) Eight miRNAs that significantly down-regulated in both CP plasma- and salivary-exosomal samples.**								

**miRNAs**	**Plasma-exosomal samples**				**Salivary-exosomal samples**			
	**Corrected *p*-value**	**FC**	**r (*p*-value)**	**AUC**	**Corrected *p*-value**	**FC**	**AUC**	**r (*p*-value)**

hsa-let-7d-5p	1.61E-04	−26.76	−0.728 (0.040)	1.00	3.94E-09	−80.30	0.50	NA
hsa-miR-103a-3p	3.55E-04	−33.96	−0.765 (0.076)	1.00	3.94E-09	−80.30	0.50	NA
hsa-miR-126-3p	2.98E-04	−24.02	−0.723 (0.043)	1.00	3.94E-09	−80.30	0.50	NA
hsa-miR-150-5p	5.17E-03	−17.14	-0.424 (0.295)	0.89	3.94E-09	−80.30	0.50	0.462 (0.250)
hsa-miR-199a-3p	1.84E-04	−22.94	-0.731 (0.039)	1.00	3.94E-09	−80.30	0.50	NA
hsa-miR-4485-5p	1.45E-03	−47.05	−0.668 (0.070)	1.00	9.54E-03	−372.05	0.67	0.243 (0.562)
hsa-miR-6088	3.11E-04	−42.38	−0.553 (0.155)	1.00	1.74E−03	−32.94	0.22	0.406 (0.318)
hsa-miR-6821-5p	5.91E-05	−54.17	−0.639 (0.088)	1.00	9.52E-04	−29.21	0.11	0.539 (0.168)

*FC = fold-change; AUC = area under curve. Negative FC value represents down-regulation, whereas positive FC value represents up-regulation. r = Pearson’s correlation coefficients. NA = not available because one of the variable (total gene signal) for all CP and healthy samples are the same [cannot be computed].*

### Bioinformatics

Total predicted target genes for each selected miRNA (from Tables 2A–C) were more than 200. Each of the miRNAs produces different set of overlap inflammation-related target genes (Table 3).

**TABLE 3:** Total of all predicted genes (predicted by TargetScan) for top ten (according to FC value) of up- and down-regulated miRNAs in CP plasma- and salivary-exosomal samples.

miRNAs	Total predicted target	Predicted inflammation-related target genes
hsa-let-7b-5p	1191	BCL2L1, COL1A2, TNFAIP3, SOCS1, STAT3, TGFBR1
hsa-miR-1224-5p	207	FGF1, NFIB
hsa-miR-125a-3p	4649	FGF1, FGF7, FGFR2, IL1R1, IL11, IL17F, MAPK9, NFIB, PPIA, RIPK1, SOCS3, SOCS5, TIMP3, TLR4
hsa-miR-1287-5p	3858	BCL2L11, BMPR2, FADD, FGFR2, NFIB, STAT3, TLR4
hsa-miR-208a-5p	4774	BCL2L11, BCL2L1, NFIB, PPIA, PTX3, SOCS5, SPRED1, TIMP3, TGFBR1, TLR4, TRAF6, VCAM1
hsa-miR-21-5p	382	BCL2, BCL11B, BMPR2, FGF7, NFIB, STAT3, TIMP3
hsa-miR-24-3p	741	BCL2L11, BCL2L2, IL1R1
hsa-miR-320a/320d	842	TGFBR1
hsa-miR-3663-3p	3024	BCL11B, BCL2L11, MAPK9, MMP13, NFKB1, SOCS1, SOCS3, TGFBR1, TLR4
hsa-miR-378i	214	BCL2L2
hsa-miR-4440	613	BCL2
hsa-miR-4485-3p	527	TLR4
hsa-miR-4485-5p	2117	BCL2L2, BMPR2, FADD, MAPK9, NFIB, SOCS3, STAT3
hsa-miR-4664-3p	629	BMPR2
hsa-miR-4793-3p	5007	BCL2L1, BCL2, BCL2L11, FADD, FGF1, IL11, NFIB, SOCS1, SOCS5, RIPK1, TLR4, VCAM1, VEGFA
hsa-miR-5006-5p	4496	BCL11B, BCL2L2, BMPR2, IL1R1, IL8, IL11, TNFAIP3, NFKB1, RIPK1, SPRED1, STAT3, TRAF6
hsa-miR-575	4425	BCL2L1, BCL2, BMPR2, FGF1, FGFR2, NFKBR1, SOCS3, SOCS5, SPRED1, STAM, STAT3, TIMP3, TLR4, TRAF6, VCAM1
hsa-miR-6073	2373	BCL2L1, IRAK1, NFIB, SPRED1, TGFBR1, TLR4, TRAF6
hsa-miR-6088	487	BCL2, BMPR2, FGF1
hsa-miR-629-3p	5848	BCL11B, BCL2L11, BCL2, BCL2L2, BMPR2, FGFR2, IL17F, MMP13, NFIB, SOCS3, SOCS5, SPRED1, TIMP3, TGFBR1, TRAF6
hsa-miR-6786-5p	1214	IL1R1, NFIB
hsa-miR-6821-5p	1106	FGFR2, MAPK9, RIPK1, TRAF6
hsa-miR-6824-5p	2531	BCL11B, BCL2L1, TNFAIP3, NFIB, TLR4
hsa-miR-6850-5p	545	BCL2, BCL11B, SOCS5
hsa-miR-7108-5p	3589	BCL11B, BCL2, BMPR2, FGF7, FGFR2, IRAK1, MAPK9, NFIB, SCOS5,TRAF6
hsa-miR-92a-3p	1037	BCL2L11, BCL11B, BMPR2, COL1A2, NFIB, SOCS5
hsa-miR-936	3187	BCL11B, FGF1, FGF7, PTX3, SPRED1, TLR4
hsa-miR-942-3p	3853	BCL2L11, BMPR2, FGF1, IL1R1, TGFBR1, TLR4, TNF

*These predicted genes that overlap with predicted inflammation-related genes in previous CP studies are short-listed as below. BCL2 = B-cell lymphoma 2; BCL11B = B-cell lymphoma 11B; BCL2L1 = B-cell lymphoma 2 like 1; BCL2L2 = B-cell lymphoma 2 like 2; BCL2L11 = B-cell lymphoma 2 like 11; BMPR2 = bone morphogenetic protein receptor type 2; COL1A2 = collagen type 1 alpha 2 chain; FADD = Fas-associated death domain; FGF1 = fibroblast growth factor 1; FGF2 = fibroblast growth factor 2; FGF7 = fibroblast growth factor 7; FGFR2 = fibroblast growth factor receptor 2; IL8 = interleukin 8; IL11 = interleukin 11; IL17F = interleukin 17F; IL1R1 = interleukin 1 receptor type 1; IRAK1 = interleukin 1 receptor associated kinase 1; MAPK9 = mitogen-activated protein kinase 9; MMP13 = matrix metallopeptidase 13; NFIB = nuclear factor 1B; nuclear factor of kappa light polypeptide gene enhancer in B-cells 1; PPIA = peptidylprolyl isomerase A; PTX3 = pentraxin 3; RIPK1 = receptor interacting protein kinase 1; SOCS1 = suppressor of cytokine signaling 1; SOCS3 = suppressor of cytokine signaling 3; SOCS5 = suppressor of cytokine signaling 5; SPRED1 = sprouty-related EVH1 domain containing 1; STAM = signal transducing adaptor molecule (SH3 domain and ITAM motif) 1; STAT3 = signal transducing and activator of transcription 3; TIMP3 = tissue inhibitors of metalloproteinases-3; TGFBR1 = transforming growth factor beta 1; TLR4 = toll-like receptor 4; TNF = tumor necrosis factor; TNFAIP3 = TNF alpha induced protein 3; TRAF6 = TNF receptor associated factor 6; VCAM1 = vascular cell adhesion molecule1; VEGFA = vascular endothelial growth factor A.*

### Evaluation the Selected MiRNAs as Potential Biomarker

Receiver Operating Characteristics curves were used to examine the discriminatory power of the listed miRNAs in Table 2, as potential biomarkers for CP as compared to healthy samples (Supplementary Data 1). The ROC curves are representative of the sensitivity (true positive rate (*Y*-axis) and one-specificity (false-positive rate: *X*-axis), while the AUC value (Table 2) indicates the discriminatory power of the biomarkers. From the Table 2A, from 33 significantly down-regulated miRNAs in CP plasma-exosomal samples, all except hsa-miR-3663-3p (AUC = 0.83), hsa-miR-25-3p (AUC = 0.5), and hsa-miR-150-5b (AUC = 0.89), had AUC value of 1. Meanwhile, all 10 significantly up-regulated miRNAs in CP salivary-exosomal samples, except hsa-miR-575 (AUC = 0.67) and hsa-miR-4793-3p (AUC = 0.83) had AUC = 1. However, the top ten significantly down-regulated miRNAs in CP salivary-exosomal samples only managed to get AUC value around 0.47–0.67 (Table 2B). The 8 miRNAs that significantly down-regulated in both CP plasma- and salivary-exosomal samples have their AUC values differently in both samples: hsa-let-7d-5p (AUC = 1 [plasma]/0.5 [saliva]), hsa-miR-103a-3p (AUC = 1 [plasma]/0.5 [saliva]), hsa-miR-126-3p (AUC = 1 [plasma]/0.5 [saliva]), hsa-miR-150-5p (AUC = 0.89 [plasma]/0.5 [saliva]), hsa-miR-199a-3p (AUC = 1 [plasma]/0.5 [saliva]), hsa-miR-4485-5p (AUC = 1 [plasma]/0.67 [saliva]), hsa-miR-6088 (AUC = 1 [plasma]/0.22 [saliva]) and hsa-miR-6821-5p (AUC = 1 [plasma]/0,11 [saliva]) (Table 2C). These showed that significantly down-regulated miRNAs from CP plasma-exosomal samples are having better AUC value, which lead to be better potential biomarker for CP as compared to down-regulated miRNAs from CP salivary-exosomal samples. In addition to that, all up-regulated miRNAs (except for hsa-miR-575) from CP salivary-exosomal samples can also be used for potential biomarker for CP as their AUC values are all above 0.8.

A Pearson’s correlation coefficient was calculated on 8 miRNAs that significantly down-regulated in both CP plasma- and salivary-exosomal samples; together with CP salivary-exosomal hsa-miR-125a-3p and hsa-miR320a; as they have showed similar expression in more than 1 source of samples, and having AUC more than 0.7. The correlation was calculated using total gene signal and clinical parameter-i.e., mean of PPD. There is significant negative strong correlation between the total gene signal of CP plasma-exosomal hsa-let-7d-5p (*r* = −0.728; *p*-value = 0.04), hsa-miR-126-3p (*r* = −0.723; *p*-value = 0.043) and hsa-miR-199a-3p (*r* = −0.731; *p*-value = 0.039) (Table 2C). Meanwhile only hsa-miR-125a-3p from CP salivary-exosomal samples showed a strong positive correlation (*r* = 0.906; *p*-value = 0.02). Other tested miRNAs, although some might showed a strong correlation, but they are yet not significant.

## Discussion

Success in exosomes isolation was proven by the detection of exosomes markers CD9, CD63, Hsp70, and CD81 (tetraspanins). Tetraspanins can interact with integrins, leading to the integrin-mediated cell adhesion extracellular matrix, hence playing role in intracellular transport (Pols and Klumperman, 2009). Exosomes are known to play the role of intracellular transporters (Yim et al., 2016). Hence, the use of these antibodies as exosomes markers is a prompt choice. This similar detection was also reported in previous studies (Ren et al., 2011; Zlotogorski-Hurvitz et al., 2015). The detection of single exosome with size around 50–100 nm under TEM also confirmed the successful of exosomes isolation from both plasma and saliva samples.

We demonstrated here for the first time that miRNAs from the plasma- and salivary-exosomes of CP individuals could serve as a potential diagnostic biomarker. The profiling done recorded the most abundant number of miRNAs with a significant difference in expression as compared to previous CP miRNA studies. A total of 1,985 down-regulated/10 up-regulated miRNAs was recorded as compared to other studies: (1) only 28 up-regulated (Lee et al., 2011), (2) 85 down-regulated/91 up-regulated (Xie et al., 2011), (3) 68 down-regulated/91 up-regulated (Stoecklin-Wasmer et al., 2012), (4) 22 down-regulated/17 up-regulated (Ogata et al., 2014), and (5) 287 down-regulated/332 up-regulated (Saito et al., 2017). Apart from Ogata (Ogata et al., 2014), other studies (Lee et al., 2011; Xie et al., 2011; Stoecklin-Wasmer et al., 2012; Saito et al., 2017) also recorded a different pattern: had more up-regulated than down-regulated miRNAs. We speculated different source of miRNAs (GCF/tissue biopsies); different total tested miRNAs and different platform used for profiling as factors contributing to this disagreement.

This study also produces high fold change for down-regulated miRNAs. Obvious result can be seen clearly in salivary-exosomal miRNAs study: fold change in range of 200–400 folds (top 10 down-regulated miRNAs). Yet, the highest fold change reported by other studies was only up to 10 folds (Lee et al., 2011; Xie et al., 2011; Ogata et al., 2014; Saito et al., 2017). As this was a preliminary study, thus a validation by using real-time would be required in future to prove this finding. If the validation results parallel to this finding, we can conclude that saliva is the best potential biomarker for the disease (high fold change denoted easy detection).

Despite of these dissimilarities, miRNAs expression from plasma- and salivary-exosomes showed better performance in expressing constantly throughout different source of samples. (1) Eight miRNAs were down-regulated in both plasma- and salivary-exosomal samples (Table 2C). (2) The down-regulation (with FC = −80.30) of (a) hsa-miR-30e-3p (AUC = 0.50), hsa-miR-31-3p (AUC = 0.50), hsa-miR-99b-5p (AUC = 0.50), hsa-miR-210-3p (AUC = 0.50), and (b) hsa-miR-193a-3p (AUC = 0.50) in CP salivary-exosomal samples were parallel with previous findings using samples from: (a) both tissues (Stoecklin-Wasmer et al., 2012) and GCF (Saito et al., 2017), and (b) only tissues (Stoecklin-Wasmer et al., 2012; Ogata et al., 2014), respectively. (3) The up-regulation of (c) hsa-miR-575 (AUC = 0.67), (d) hsa-miR-125a-3p (AUC = 1), (e) hsa-miR-320a (AUC = 1) in CP salivary-exosomal samples were parallel with findings using samples from: (c) tissues (Stoecklin-Wasmer et al., 2012), (d) GCF (Saito et al., 2017), and (e) tissues (Stoecklin-Wasmer et al., 2012), respectively. (4) Comparing miRNAs-periodontitis studies from a similar source (tissues samples) (Xie et al., 2011; Stoecklin-Wasmer et al., 2012; Ogata et al., 2014), only hsa-miR-223 has similar expression (up-regulated) in periodontitis samples (fold change 1.5 to more than 2). Hence, these findings prove plasma- and salivary-exosomes are reliable source for miRNAs studies.

The ROC curves and AUC value for tabulated miRNAs were built and calculated as one of other step to prove the potential of these miRNAs to be developed as biomarkers. Unfortunately, AUC value of top ten significantly down-regulated miRNAs in CP salivary-exosomal samples only showed in the range of 0.5–0.67, which close to useless value. The guidelines for interpreting the AUC values noted that an AUC value at 0.5 or less cannot discriminate between the two condition; AUC of 0.51–0.69 shows unacceptably low discrimination; AUC of 0.7–0.79 as acceptable discriminatory; AUC of 0.8–0.89 as excellent discriminatory and AUC of 0.9-1 as an outstanding discriminatory (Looney and Hagan, 2015). Table 2B showed contradict conclusion between the fold change and AUC value. The most down-regulated miRNAs (by fold change value) that supposedly having a good AUC, only recorded AUC with unacceptably low discriminatory power. However, we speculated that a very low samples size might affect the outcome (Bujang and Adnan, 2016). Thus, these findings need to be confirmed with AUC from validation step (usually by qPCR, using relative expression to calculate AUC), with more samples, as done by Yoneda et al. (2019) and Han et al. (2020).

Table 3 shows more than 200 target genes were predicted by TargetScan, for each selected miRNA. The overlapping process shows that each miRNA has a different set of inflammation-related target genes (Table 3). From this list, toll-like receptor (TLR) relationship with CP disease is described here. Previous DNA microarray study has shown an up-regulated expression of toll-like receptor 4 (TLR4) in periodontitis patients’ gingival fibroblasts as compared to healthy individuals (Wang et al., 2003). TLR functions in recognizing and distinguishing highly conserved structures present in microorganisms (Mahanonda and Pichyangkul, 2007). Binding of ligand to the TLR signals activates signal transduction, leading to transcription of pro-inflammatory cytokines, which initiate the immune responses. However, too much production of cytokines can lead to the destruction of connective tissue and bone (leading to chronic inflammation and bone loss: common feature in periodontics diseases). TLR4 recognizes lipopolysaccharides from both two key periodontal pathogens- the *Porphyromonas gingivalis* (Darveau et al., 2004), and *Actinobacillus actinomycetemcomitans* (Mochizuki et al., 2004) as ligands. The up-regulation of hsa-miR-125a-3p, hsa-miR-4793-3p, hsa-miR-575, and hsa-miR-6824-5p; and down-regulation of hsa-miR-1287, hsa-miR-208a-5p, hsa-miR-3663-3p, hsa-miR-4485-3p, hsa-miR-6073, hsa-miR-936 and hsa-miR-942-3p in CP samples suggest their roles in targeting the TLF4, which is similar to those reported in previous studies (Wu et al., 2014; Tan et al., 2018; Xu et al., 2019). If we consider the calculated AUC values, except for hsa-miR-4485-3p, hsa-miR-6073, hsa-miR-936 and hsa-miR-942-3p (all from CP salivary-exosomal samples), other 6 miRNAs are worthy for future study in confirming whether they are correctly targeting TLR4; and to construct the relationship between miRNAs-TLR4-periodontitis. These miRNAs might be functioning like miR-146a: as a negative feedback regulator (down-regulates pro-inflammatory cytokine secretion and blocks TLR4 signaling in CP cell line mimic) (Jiang et al., 2015).

## Conclusion

In conclusion, we have explored miRNAs expression patterns in both plasma- and salivary-exosomes. Thirty-three miRNAs were identified as differentially expressed more than 2-fold in plasma-exosomes, whereas 1,995 miRNAs in salivary-exosomes. The ROC curves were built and AUC values were calculated to predict the discriminatory power of the miRNAs. These differential miRNAs were then further analyzed using TargetScan to predict their respective target genes. Although no validation (by real-time PCR) and functional studies were done, present data managed to highlight some miRNAs that have the potential to be a signature and biomarker candidate for the CP disease. In future, we plan to continue this study with (1) validation step by quantitative real-time PCR method (using calculated sample size), and (2) Regulatory mechanisms of miRNA by using luciferase reporter assay (that might explain the function of selected miRNAs in chronic periodontitis disease). Using plasma-exosomal samples, hsa-miR-let-7d [FC = −26.76; AUC = 1; *r* = −0.728 [*p*-value = 0.04]), hsa-miR-126-3p (FC = −24.02; AUC = 1; *r* = −0.723 [*p*-value = 0.043]) and hsa- hsa-miR-199a-3p (FC = −22.94; AUC = 1; *r* = −0.731 [*p*-value = 0.039]) are worth to be furthered studied. Using salivary-exosomal samples, hsa-miR-125a-3p (FC = 2.03; AUC = 1; *r* = 0.91 [*p*-value = 0.02]) is worth to be furthered studied. These miRNAs can be concluded to be the reliable candidates for the development of periodontitis biomarker, as they were significantly expressed differently, with a good discriminatory value and strongly correlate with the mean of PPD.

## Data Availability Statement

The raw data supporting the conclusions of this article will be made available by the authors, without undue reservation.

## Ethics Statement

The studies involving human participants were reviewed and approved by the Human Research Ethics Committee of USM (USM/JEPeM/16100397). The patients/participants provided their written informed consent to participate in this study.

## Author Contributions

NN, SM, and WS designed the study. NN recruited individuals for the study. RR validated the clinical examination of recruited individuals for the study. RR helped NN in writing the first draft of the manuscript, which was critically revised by SM and WS. All authors approved the final version of the manuscript, and agreed to be accountable for all aspects of the work.

## Conflict of Interest

The authors declare that the research was conducted in the absence of any commercial or financial relationships that could be construed as a potential conflict of interest.

## Footnotes

1 http://www.targetscan.org/vert_71/

## Supplementary Material

Supplementary Figure 1 Electropherograms of CP and healthy samples. Representative electropherograms of total RNA samples were derived from the plasma of CP **(A,B)** and healthy **(C,D)**. Representative electropherograms of total RNA samples were derived from the saliva of CP **(E,F)** and healthy **(G,H)**. For RNA Nano 6000 chip **(A,C,E,F)**, peaks of small RNAs whose size was less than 200 nt (nucleotides) were observed at 25 s (seconds) or between peak for markers 25 and 200 nt. For small RNA chip **(B,D,F,H)**, peaks of miRNAs whose size less than 30 nt were observed at 20 nt. [FU = fluorescence unit].

Supplementary Data 1 Receiver Operating Characteristics (ROC) curves. ROC curves of all 33 significantly down-regulated miRNAs in CP plasma-exosomal samples as compared to healthy plasma-exosomal samples; together with their area under curve table **(1)**. ROC curves of all 10 significantly up-regulated miRNAs in CP salivary-exosomal samples as compared to healthy salivary-exosomal samples; together with their area under curve table **(2)**. ROC curves of top ten significantly down-regulated miRNAs in CP salivary-exosomal samples as compared to healthy salivary-exosomal samples; together with their area under curve table **(3)**. ROC curves of eight miRNAs that significantly down-regulated miRNAs in both CP plasma- and salivary-exosomal samples as compared to healthy plasma- and salivary-exosomal samples, respectively; together with their area under curve table **(4)**. ROC curves of significantly down-regulated miRNAs in CP salivary-exosomal samples as compared to healthy salivary-exosomal samples, and parallel with previous CP studies’ findings; together with their area under curve table **(5)**. ROC curves of significantly up-regulated miRNAs in CP salivary-exosomal samples as compared to healthy salivary-exosomal samples, and parallel with previous CP studies’ findings; together with their area under curve table **(6)**. All the ROC curves with AUC = 1 have significant *p*-value (*p*-value = 0.05), while ROC curves with AUC value < 1 does not have significant *p*-value (*p*-value > 0.05).
