# Supplementary figures and images for: Plasma- and Saliva Exosome Profile Reveals a Distinct MicroRNA Signature in Chronic Periodontitis
Source: Front Physiol. 2020 Nov 30;11:587381. doi: 10.3389/fphys.2020.587381 (PMC7733931; doi:10.3389/fphys.2020.587381)

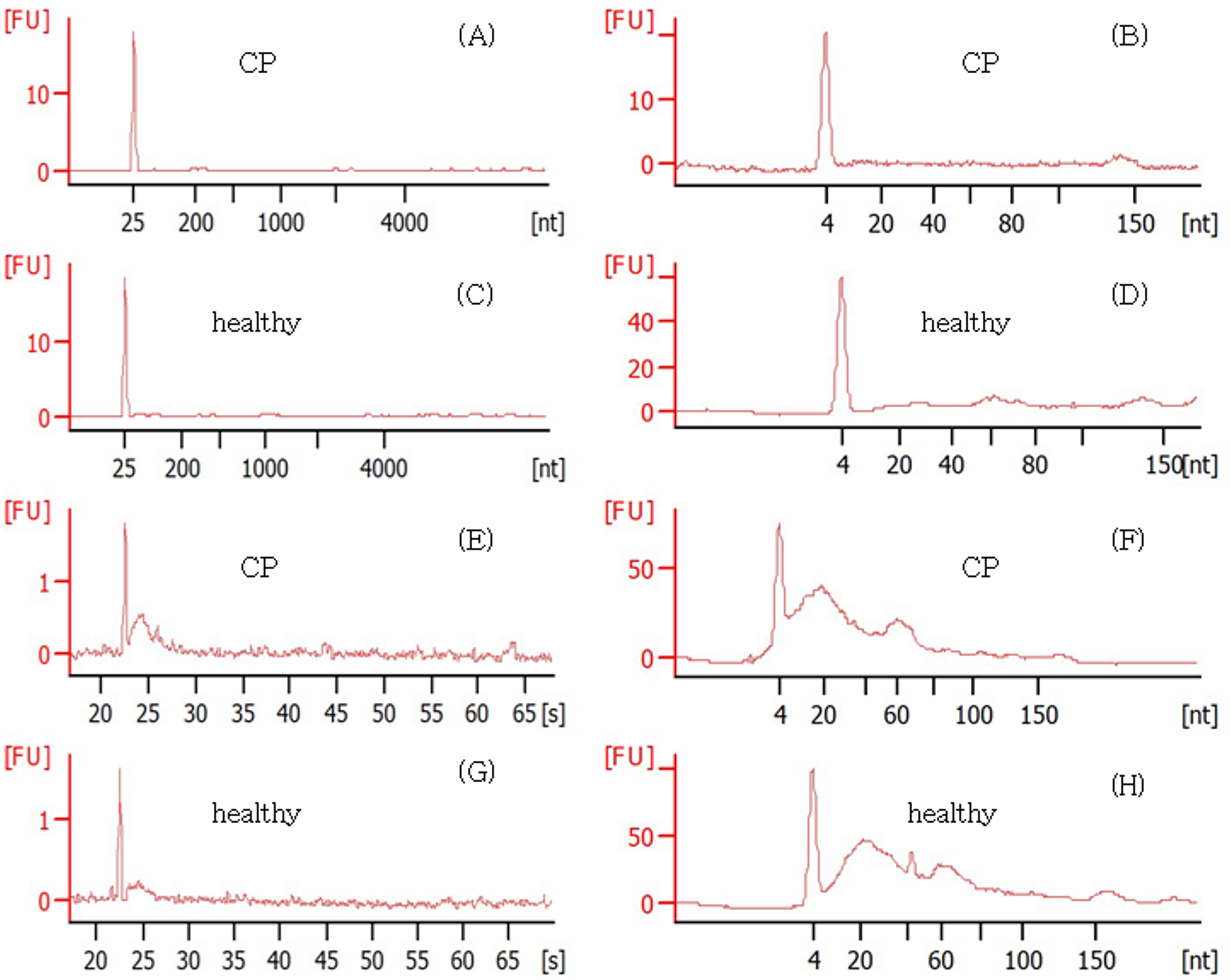

Supplement: Supplementary Figure 1 — Electropherograms of CP and healthy samples. Representative electropherograms of total RNA samples were derived from the plasma of CP (A,B) and healthy (C,D). Representative electropherograms of total RNA samples were derived from the saliva of CP (E,F) and healthy (G,H). For RNA Nano 6000 chip (A,C,E,F), peaks of small RNAs whose size was less than 200 nt (nucleotides) were observed at 25 s (seconds) or between peak for markers 25 and 200 nt. For small RNA chip (B,D,F,H), peaks of miRNAs whose size less than 30 nt were observed at 20 nt. [FU = fluorescence unit]. [file Image_1.TIF]
