# Supplementary material for: Plasma- and Saliva Exosome Profile Reveals a Distinct MicroRNA Signature in Chronic Periodontitis
Source: Front Physiol. 2020 Nov 30;11:587381. doi: 10.3389/fphys.2020.587381 (PMC7733931; doi:10.3389/fphys.2020.587381)
Supplement: Supplementary Data 1 — Receiver Operating Characteristics (ROC) curves. ROC curves of all 33 significantly down-regulated miRNAs in CP plasma-exosomal samples as compared to healthy plasma-exosomal samples; together with their area under curve table (1). ROC curves of all 10 significantly up-regulated miRNAs in CP salivary-exosomal samples as compared to healthy salivary-exosomal samples; together with their area under curve table (2). ROC curves of top ten significantly down-regulated miRNAs in CP salivary-exosomal samples as compared to healthy salivary-exosomal samples; together with their area under curve table (3). ROC curves of eight miRNAs that significantly down-regulated miRNAs in both CP plasma- and salivary-exosomal samples as compared to healthy plasma- and salivary-exosomal samples, respectively; together with their area under curve table (4). ROC curves of significantly down-regulated miRNAs in CP salivary-exosomal samples as compared to healthy salivary-exosomal samples, and parallel with previous CP studies’ findings; together with their area under curve table (5). ROC curves of significantly up-regulated miRNAs in CP salivary-exosomal samples as compared to healthy salivary-exosomal samples, and parallel with previous CP studies’ findings; together with their area under curve table (6). All the ROC curves with AUC = 1 have significant p-value (p-value = 0.05), while ROC curves with AUC value < 1 does not have significant p-value (p-value > 0.05). [file Table_1.DOCX]

Supplementary Data 1

**[Receiver operating characteristic (ROC) curves](https://en.wikipedia.org/wiki/Receiver_operating_characteristic)**

1. ROC curves of all 33 significantly down-regulated miRNAs in CP plasma exosomal samples as compared to healthy plasma exosomal samples:


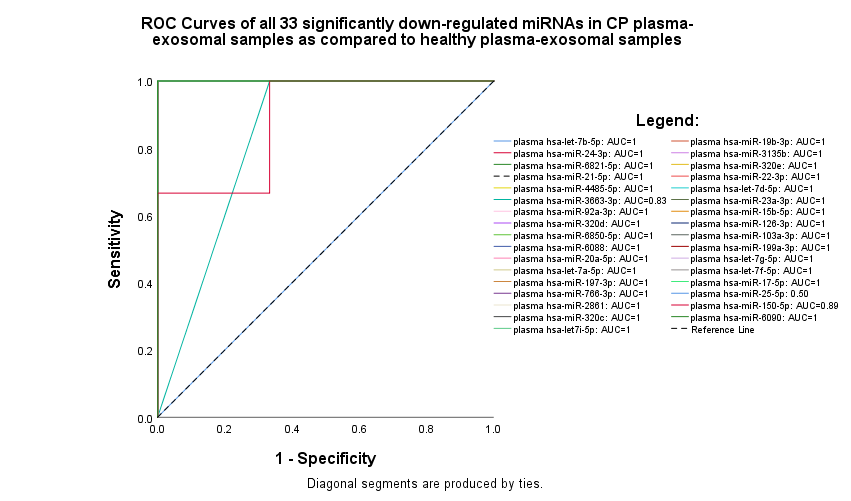


| **Area Under the Curve** | | | | | |
| --- | --- | --- | --- | --- | --- |
| Test Result Variable(s) | Area | Std. Error^a^ | Asymptotic Sig.^b^ | Asymptotic 95% Confidence Interval | |
|  |  |  |  | Lower Bound | Upper Bound |
| plasma_hsalet7b_5p | 1.000 | .000 | .050 | 1.000 | 1.000 |
| plasma_hsamiR24_3p | 1.000 | .000 | .050 | 1.000 | 1.000 |
| plasma_hsamiR6821_5p | 1.000 | .000 | .050 | 1.000 | 1.000 |
| plasma_hsamiR21_5p | 1.000 | .000 | .050 | 1.000 | 1.000 |
| plasma_hsamiR4485_5p | 1.000 | .000 | .050 | 1.000 | 1.000 |
| plasma_hsamiR3663_3p | .833 | .190 | .190 | .461 | 1.000 |
| plasma_hsamiR92a_3p | 1.000 | .000 | .050 | 1.000 | 1.000 |
| plasma_hsamiR320d | 1.000 | .000 | .050 | 1.000 | 1.000 |
| plasma_hsamiR6850_5p | 1.000 | .000 | .050 | 1.000 | 1.000 |
| plasma_hsamiR6088 | 1.000 | .000 | .050 | 1.000 | 1.000 |
| plasma_hsamiR20a_5p | 1.000 | .000 | .050 | 1.000 | 1.000 |
| plasma_let7a_5p | 1.000 | .000 | .050 | 1.000 | 1.000 |
| plasma_hsamiR197_3p | 1.000 | .000 | .050 | 1.000 | 1.000 |
| plasma_hsamiR766_3p | 1.000 | .000 | .050 | 1.000 | 1.000 |
| plasma_hsamiR2861 | 1.000 | .000 | .050 | 1.000 | 1.000 |
| plasma_hsamiR320c | 1.000 | .000 | .050 | 1.000 | 1.000 |
| plasma_let7i_5p | 1.000 | .000 | .050 | 1.000 | 1.000 |
| plasma_hsamiR19b_3p | 1.000 | .000 | .050 | 1.000 | 1.000 |
| plasma_hsamiR3135b | 1.000 | .000 | .050 | 1.000 | 1.000 |
| plasma_hsamiR320e | 1.000 | .000 | .050 | 1.000 | 1.000 |
| plasma_hsamiR22_3p | 1.000 | .000 | .050 | 1.000 | 1.000 |
| plasma_let7d_5p | 1.000 | .000 | .050 | 1.000 | 1.000 |
| plasma_hsamiR23a_3p | 1.000 | .000 | .050 | 1.000 | 1.000 |
| plasma_hsamiR15b_5p | 1.000 | .000 | .050 | 1.000 | 1.000 |
| plasma_hsamiR126_3p | 1.000 | .000 | .050 | 1.000 | 1.000 |
| plasma_hsamiR103a_3p | 1.000 | .000 | .050 | 1.000 | 1.000 |
| plasma_hsamiR199a_3p | 1.000 | .000 | .050 | 1.000 | 1.000 |
| plasma_let7g_5p | 1.000 | .000 | .050 | 1.000 | 1.000 |
| plasma_let7f_5p | 1.000 | .000 | .050 | 1.000 | 1.000 |
| plasma_hsamiR17_5p | 1.000 | .000 | .050 | 1.000 | 1.000 |
| plasma_hsamiR25_5p | .500 | .255 | 1.000 | .001 | .999 |
| plasma_hsamiR150_5p | .889 | .148 | .127 | .599 | 1.000 |
| plasma_hsamiR6090 | 1.000 | .000 | .050 | 1.000 | 1.000 |
| The test result variable(s): plasma_hsamiR3663_3p, plasma_hsamiR25_5p has at least one tie between the positive actual state group and the negative actual state group. Statistics may be biased. | | | | | |
| a. Under the nonparametric assumption | | | | | |
| b. Null hypothesis: true area = 0.5 | | | | | |

1. ROC curves of all 10 significantly up-regulated miRNAs in CP salivary exosomal samples as compared to healthy salivary exosomal samples:


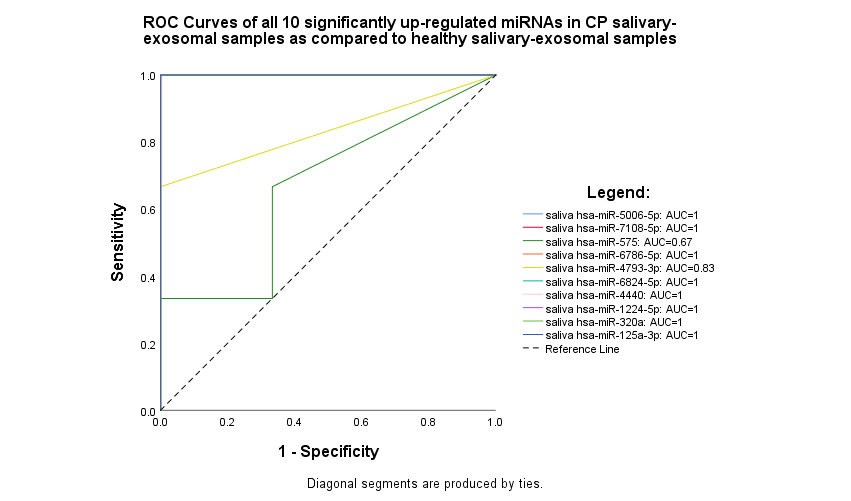


| **Area Under the Curve** | | | | | |
| --- | --- | --- | --- | --- | --- |
| Test Result Variable(s) | Area | Std. Error^a^ | Asymptotic Sig.^b^ | Asymptotic 95% Confidence Interval | |
|  |  |  |  | Lower Bound | Upper Bound |
| saliva_hsamiR5006_5p | 1.000 | .000 | .050 | 1.000 | 1.000 |
| saliva_hsamiR7108_5p | 1.000 | .000 | .050 | 1.000 | 1.000 |
| saliva_hsamiR575 | .667 | .240 | .513 | .196 | 1.000 |
| saliva_hsamiR6786_5p | 1.000 | .000 | .050 | 1.000 | 1.000 |
| saliva_hsamiR4793_3p | .833 | .190 | .190 | .461 | 1.000 |
| saliva_hsamiR6824_5p | 1.000 | .000 | .050 | 1.000 | 1.000 |
| saliva_hsamiR4440 | 1.000 | .000 | .050 | 1.000 | 1.000 |
| saliva_hsamiR1224_5p | 1.000 | .000 | .050 | 1.000 | 1.000 |
| saliva_hsamiR320a | 1.000 | .000 | .050 | 1.000 | 1.000 |
| saliva_hsamiR125a_3p | 1.000 | .000 | .050 | 1.000 | 1.000 |
| The test result variable(s): saliva_hsamiR575, saliva_hsamiR4793_3p has at least one tie between the positive actual state group and the negative actual state group. Statistics may be biased. | | | | | |
| a. Under the nonparametric assumption | | | | | |
| b. Null hypothesis: true area = 0.5 | | | | | |

1. ROC curves of top 10 significantly down-regulated miRNAs in CP salivary exosomal samples as compared to healthy salivary exosomal samples:


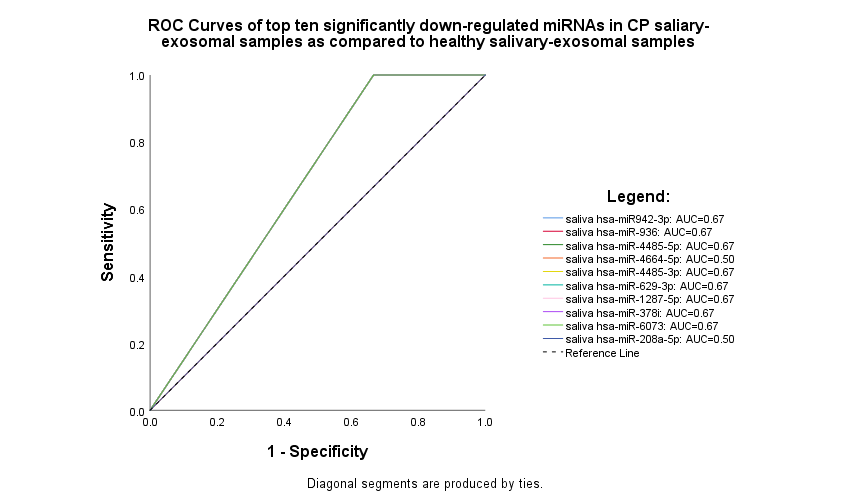


| **Area Under the Curve** | | | | | |
| --- | --- | --- | --- | --- | --- |
| Test Result Variable(s) | Area | Std. Error^a^ | Asymptotic Sig.^b^ | Asymptotic 95% Confidence Interval | |
|  |  |  |  | Lower Bound | Upper Bound |
| saliva_hsamiR942_3p | .667 | .240 | .513 | .196 | 1.000 |
| saliva_hsamiR936 | .667 | .240 | .513 | .196 | 1.000 |
| saliva_hsamiR4485_5p | .667 | .240 | .513 | .196 | 1.000 |
| saliva_hsamiR4664_5p | .500 | .255 | 1.000 | .001 | .999 |
| saliva_hsamiR4485_3p | .667 | .240 | .513 | .196 | 1.000 |
| saliva_hsamiR629_3p | .667 | .240 | .513 | .196 | 1.000 |
| saliva_hsamiR1287_5p | .667 | .240 | .513 | .196 | 1.000 |
| saliva_hsamiR378i | .667 | .240 | .513 | .196 | 1.000 |
| saliva_hsamiR6073 | .667 | .240 | .513 | .196 | 1.000 |
| saliva_hsamiR208a_5p | .500 | .255 | 1.000 | .001 | .999 |
| The test result variable(s): saliva_hsamiR942_3p, saliva_hsamiR936, saliva_hsamiR4485_5p, saliva_hsamiR4664_5p, saliva_hsamiR4485_3p, saliva_hsamiR629_3p, saliva_hsamiR1287_5p, saliva_hsamiR378i, saliva_hsamiR6073, saliva_hsamiR208a_5p has at least one tie between the positive actual state group and the negative actual state group. Statistics may be biased. | | | | | |
| a. Under the nonparametric assumption | | | | | |
| b. Null hypothesis: true area = 0.5 | | | | | |

1. ROC curves of 8 miRNAs that significantly down-regulated in both CP plasma and salivary exosomal samples as compared to healthy plasma and salivary salivary exosomal samples, respectively:


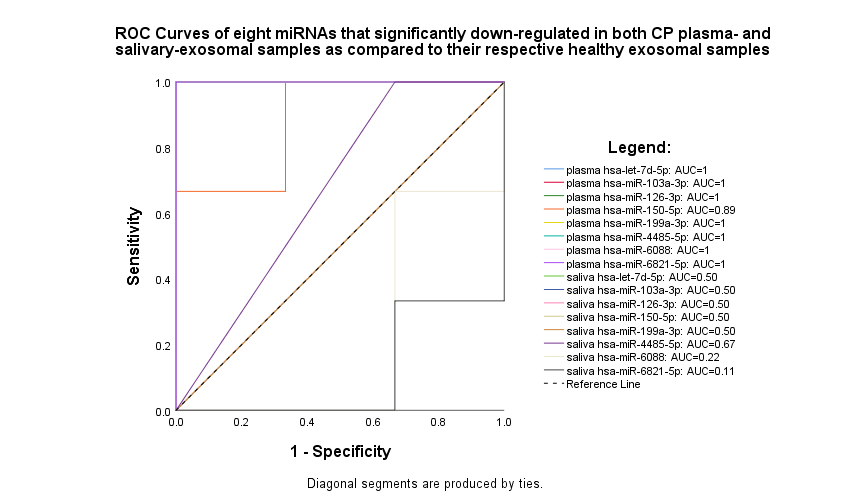


| **Area Under the Curve** | | | | | |
| --- | --- | --- | --- | --- | --- |
| Test Result Variable(s) | Area | Std. Error^a^ | Asymptotic Sig.^b^ | Asymptotic 95% Confidence Interval | |
|  |  |  |  | Lower Bound | Upper Bound |
| plasma_hsalet7d_5p | 1.000 | .000 | .050 | 1.000 | 1.000 |
| plasma_hsamiR103a_3p | 1.000 | .000 | .050 | 1.000 | 1.000 |
| plasma_hsamiR126_3p | 1.000 | .000 | .050 | 1.000 | 1.000 |
| plasma_hsamiR150_5p | .889 | .148 | .127 | .599 | 1.000 |
| plasma_hsamiR199a_3p | 1.000 | .000 | .050 | 1.000 | 1.000 |
| plasma_hsamiR4485_5p | 1.000 | .000 | .050 | 1.000 | 1.000 |
| plasma_hsamiR6088 | 1.000 | .000 | .050 | 1.000 | 1.000 |
| plasma_hsamiR6821_5p | 1.000 | .000 | .050 | 1.000 | 1.000 |
| saliva_hsalet7d_5p | .500 | .255 | 1.000 | .001 | .999 |
| saliva_hsamiR103a_3p | .500 | .255 | 1.000 | .001 | .999 |
| saliva_hsamiR126_3p | .500 | .255 | 1.000 | .001 | .999 |
| saliva_hsamiR150_5p | .500 | .255 | 1.000 | .001 | .999 |
| saliva_hsamiR199a_3p | .500 | .255 | 1.000 | .001 | .999 |
| saliva_hsamiR4485_5p | .667 | .240 | .513 | .196 | 1.000 |
| saliva_hsamiR6088 | .222 | .216 | .275 | .000 | .645 |
| saliva_hsamiR6821_5p | .111 | .148 | .127 | .000 | .401 |
| The test result variable(s): saliva_hsalet7d_5p, saliva_hsamiR103a_3p, saliva_hsamiR126_3p, saliva_hsamiR150_5p, saliva_hsamiR199a_3p, saliva_hsamiR4485_5p has at least one tie between the positive actual state group and the negative actual state group. Statistics may be biased. | | | | | |
| a. Under the nonparametric assumption | | | | | |
| b. Null hypothesis: true area = 0.5 | | | | | |

1. ROC curves of significantly down-regulated miRNAs in CP salivary exosomal samples as compared to healthy salivary salivary exosomal; and parallel with previous CP studies’ findings:


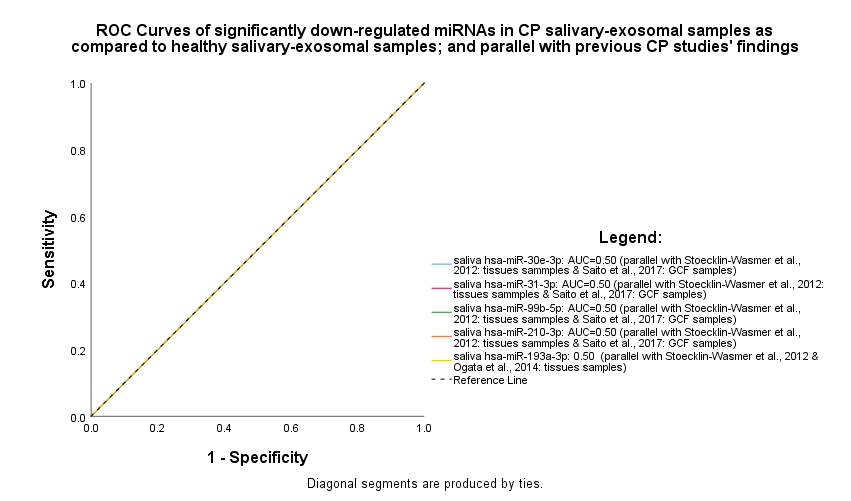


| **Area Under the Curve** | | | | | |
| --- | --- | --- | --- | --- | --- |
| Test Result Variable(s) | Area | Std. Error^a^ | Asymptotic Sig.^b^ | Asymptotic 95% Confidence Interval | |
|  |  |  |  | Lower Bound | Upper Bound |
| saliva_hsamiR30e_3p | .500 | .255 | 1.000 | .001 | .999 |
| saliva_hsamiR31_3p | .500 | .255 | 1.000 | .001 | .999 |
| saliva_hsamiR99b_5p | .500 | .255 | 1.000 | .001 | .999 |
| saliva_hsamiR210_3p | .500 | .255 | 1.000 | .001 | .999 |
| saliva_hsamiR193a_3p | .500 | .255 | 1.000 | .001 | .999 |
| The test result variable(s): saliva_hsamiR30e_3p, saliva_hsamiR31_3p, saliva_hsamiR99b_5p, saliva_hsamiR210_3p, saliva_hsamiR193a_3p has at least one tie between the positive actual state group and the negative actual state group. Statistics may be biased. | | | | | |
| a. Under the nonparametric assumption | | | | | |
| b. Null hypothesis: true area = 0.5 | | | | | |

1. ROC curves of significantly up-regulated miRNAs in CP salivary exosomal samples as compared to healthy salivary salivary exosomal; and parallel with previous CP studies’ findings:


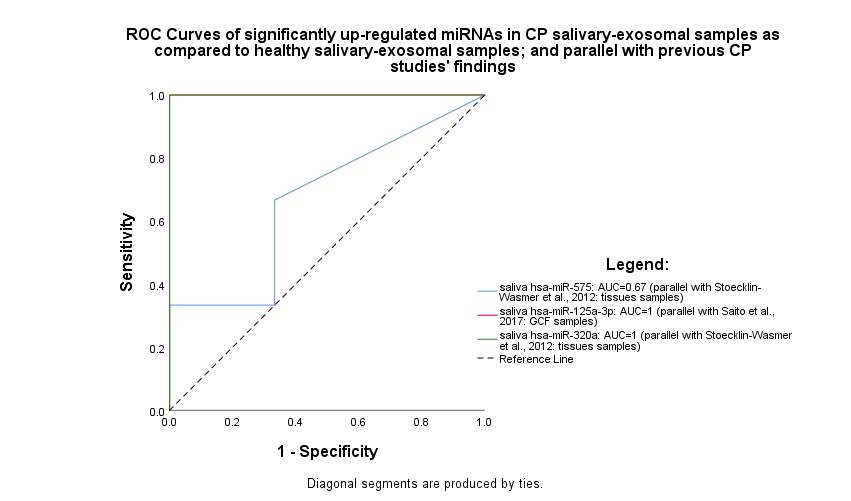


| **Area Under the Curve** | | | | | |
| --- | --- | --- | --- | --- | --- |
| Test Result Variable(s) | Area | Std. Error^a^ | Asymptotic Sig.^b^ | Asymptotic 95% Confidence Interval | |
|  |  |  |  | Lower Bound | Upper Bound |
| saliva_hsamiR575 | .667 | .240 | .513 | .196 | 1.000 |
| saliva_hsamiR125a_3p | 1.000 | .000 | .050 | 1.000 | 1.000 |
| saliva_hsamiR320a | 1.000 | .000 | .050 | 1.000 | 1.000 |
| The test result variable(s): saliva_hsamiR575 has at least one tie between the positive actual state group and the negative actual state group. Statistics may be biased. | | | | | |
| a. Under the nonparametric assumption | | | | | |
| b. Null hypothesis: true area = 0.5 | | | | | |
